# The Increased Tibiofemoral Rotation: A Potential Contributing Factor for Patellar Maltracking in Patients with Recurrent Patellar Dislocation

**DOI:** 10.1111/os.13358

**Published:** 2022-06-13

**Authors:** Guan Wu, YanWei Cao, GuanYang Song, Yue Li, Tong Zheng, Hui Zhang, ZhiJun Zhang

**Affiliations:** ^1^ Sports Medicine Service Beijing jishuitan hospital Beijing China

**Keywords:** patellar dislocation, patellar maltracking, tibial tubercle‐trochlear groove, tibiofemoral rotation

## Abstract

**Objective:**

The purpose of this study was to analyze the relationship between tibiofemoral rotation and patellar maltracking in patients with recurrent patellar dislocation.

**Methods:**

A total of 143 consecutive knees (118 patients) with clinically diagnosed recurrent patellar dislocation from January 2018 to December 2019 were retrospectively analyzed. Patellar tilt angle and bisect offset index were recorded on axial CT to assesses the severity of patellar maltracking. Tibiofemoral rotation angle is measured by comparing the angle between the posterior femoral and tibial condylar lines on three‐dimensional CT. The Pearson correlation was calculated to investigate the association between tibiofemoral rotation angle and patellar maltracking. Patients were divided into the rotation group (≥15°) and control group (<15°) based on the value of tibiofemoral rotation and a further comparison was performed. To further clarify the complicated relationship among tibial tubercle‐trochlear groove (TT‐TG), tibial tubercle‐posterior cruciate ligament distance (TT‐PCL), tibiofemoral rotation, and patellar maltracking, patients were divided into four subgroups according to the value of TT‐TG and TT‐PCL.

**Results:**

The mean preoperative tibiofemoral rotation angle was 12° ± 6° (range, 0°–31°). Pearson correlation between patellar maltracking parameters (bisect offset index, patellar tilt angle) and various bony deformities found that the tibiofemoral rotation angle was moderately correlated with bisect offset index (r = 0.451, p < 0.001) and patellar tilt angle (r = 0.462, p < 0.001). Further results demonstrated that bisect offset index (152.1 *vs* 121.2, p < 0.001) and patellar tilt angle (41.2° *vs* 33.5°, p < 0.001) were significantly higher in the rotation group than that in control group. For patients with a TT‐TG distance of >20 mm, the increased TT‐TG distance was mainly caused by tibiofemoral rotation angle in group C (TT‐TG > 20 mm, TT‐PCL < 24 mm) and predominantly induced by tibial tubercle lateralization in group D (TT‐TG > 20 mm, TT‐PCL > 24 mm). Bisect offset index and patellar tilt angle were significantly higher in the group C than group D.

**Conclusion:**

The increased tibiofemoral rotation angle is associated with patellar maltracking in patients with recurrent patellar dislocation. Patients with increased tibiofemoral rotation angle usually have more severe patellar maltracking.

## Graphical abstract

The most important finding of this study is that the increased tibiofemoral rotation angle is associated with patellar maltracking in patients with RPD. Tibiofemoral rotation‐induced TT‐TG abnormality deserves more attention in clinical practice, because these patients usually have more serious patellar maltracking compared with tibial tubercle lateralization‐caused TT‐TG abnormality.

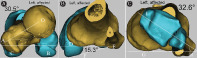

## Introduction

Patellar maltracking refers to the abnormal relationship between the patella and trochlea during knee flexion‐extension^1^, and previous studies have found that patellar maltracking is present in up to 70% of patients with recurrent patellar dislocation (RPD)^2^. J‐sign is the most commonly used visual assessment of patellar maltracking in patients with RPD^2^, ^3^. The clinical significance of patellar maltracking in the management of RPD has been validated by several studies in the literature. In a recent clinical study, Sappey *et al*. reported the clinical outcomes of 211 RPD patients treated with isolated medial patellofemoral ligament reconstruction (MPFL‐R) and found that a positive J‐sign was an independent risk factor for MPFL‐R failure^4^. Similarly, Zhang *et al*. reported that the presence of a preoperative severe patellar maltracking (high‐grade J‐sign) was associated with residual graft laxity after MPFL‐R in patients with RPD^5^. Moreover, one study analyzed the influence of the severity of patellar maltracking on clinical outcomes in patients with RPD, which found a close relationship between patellar maltracking and postoperative patient‐reported outcomes: the more severe the patellar maltracking, the worse the functional outcomes^6^. Therefore, the underlying causes of patellar maltracking should be fully clarified, which can guide the clinical treatment of RPD with patellar maltracking.

To date, it is most commonly viewed that patellar maltracking occurs as a result of an imbalance in the dynamic relationship between the patella and trochlea, which is often secondary to some underlying structural abnormalities^1^, such as muscular imbalance of the quadriceps femoris^7^, abnormal patellar height^8^, trochlear dysplasia^9^, ^10^, increased tibial tubercle‐trochlear groove (TT‐TG) distance^11^, and lower extremity rotational deformities. These deformities have been thought to increase the forced shift of the patella towards the lateral side in terminal knee extension and result in patellar maltracking.

Femoral anteversion, external tibial rotation, and tibiofemoral rotation, which are measured by comparing the angle between the posterior femoral line and tibial condylar lines and distinct from femoral or tibial torsion arising from within the respective bone, are three parameters reflecting the severity of lower extremity rotational deformities^12^, ^13^, ^14^. Many studies have found that the first two parameters (increased femoral anteversion and external tibial rotation) are contributing factors for patellar maltracking in patients with recurrent patellar dislocation^6^. In contrast, little is known about the role of tibiofemoral rotation in recurrent patellar dislocation.

Recently, tibiofemoral rotation has been identified as a potential contributing factor to recurrent patellar dislocation^12^, ^13^, ^14^, ^15^, ^16^, ^17^, ^18^. Several studies found that the tibiofemoral rotation angle was significantly higher in patients with recurrent patellar dislocation than the control group^16^, ^18^, ^19^. Some authors compared the tibiofemoral rotation angles in different populations and found that tibiofemoral rotation was correlated with the severity of patellar dislocation^13^. Further studies demonstrated that tibiofemoral rotation angle was correlated with patellar lateral shift distance in a cadaveric biomechanical study^20^. It is speculated that tibiofemoral rotation may contribute to patellar dislocation because external rotation of the tibia relative to the distal femur results in lateralization of the tibial tubercle, increased lateral tilt, attenuated medial soft tissues, and altered force vectors, all of which contribute to decreased patellar stability and patellar maltracking^13^. These above studies suggested that the tibiofemoral rotation might play an important role in the formation of patellar maltracking in patients with recurrent patellar dislocation.

To our knowledge, no studies have systematically investigated the association between increased tibiofemoral rotation and patellar maltracking in patients with recurrent patellar dislocation. Therefore, the purpose of this study was: (i) to report the value of tibiofemoral rotation angle in patients with recurrent patellar dislocation; and (ii) to analyze the relationship between tibiofemoral rotation angle and patellar maltracking. We hypothesized that the increased tibiofemoral rotation angle is a potential contributing factor for patellar maltracking in patients with recurrent patellar dislocation.

## Materials and Methods

A total of 169 patients were diagnosed with RPD and surgically treated in our institution from January 2018 to December 2019. The inclusion criteria were: (i) recurrent patellar dislocation; (ii) record of preoperative J‐sign; (iii) presence of preoperative imaging. The exclusion criteria were: (i) revision surgery; (ii) acute first‐time dislocation; (iii) lack of preoperative CT imaging. Finally, a total of 143 knees in 118 patients who met the criteria were enrolled in the study. This study was approved by our Institutional Ethics Board (IRB approval number: 01‐07‐2020).

### Patellar Maltracking Assessment

**Fig. 1:**
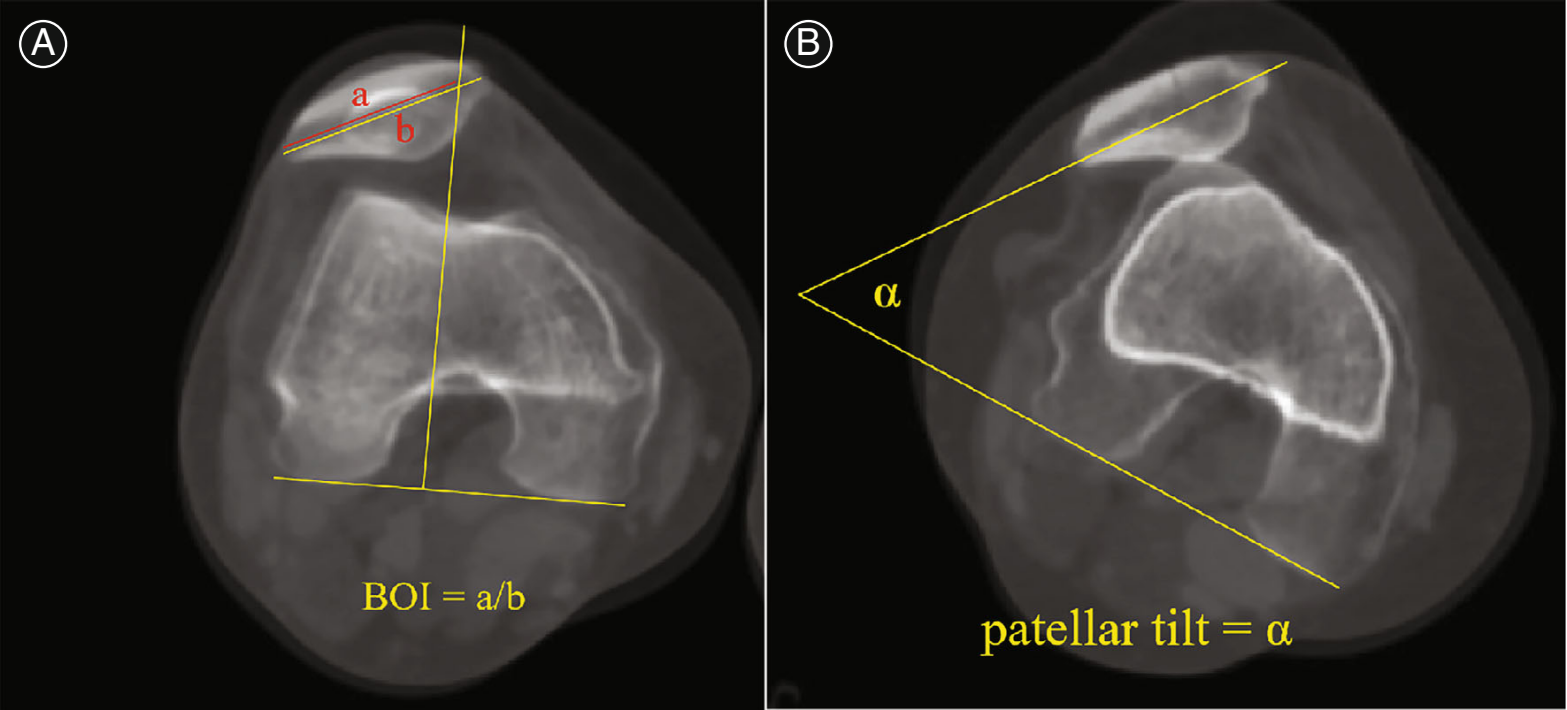
Measurement of patellar maltracking. (A), BOI measurements (a/b × 100%) were performed by measuring the percentage of the width of the patella that was lateral to a line through the deepest point of the trochlear groove. (B), patellar tilt angle measures the angle between the medial–lateral axis of the patella and the posterior condylar axis of the femur.

Patellar maltracking was characterized by the bisect offset index (BOI) and patellar tilt angle on CT^2^. BOI, which assesses medial or lateral subluxation of the patella, quantifies the normalized width of the patella lateral to the trochlear groove. The BOI is calculated by dividing the length along the patellar width from the lateral‐most point to the line through the groove by the total width of the patella. Patellar tilt angle is measured as the angle formed between the posterior condylar axis and the patellar width line (Figure 1). Moreover, knee range of motion (ROM) and J‐sign are also assessed during physical examination.

### Lower‐Leg Rotational Parameters Assessment

**Fig. 2:**
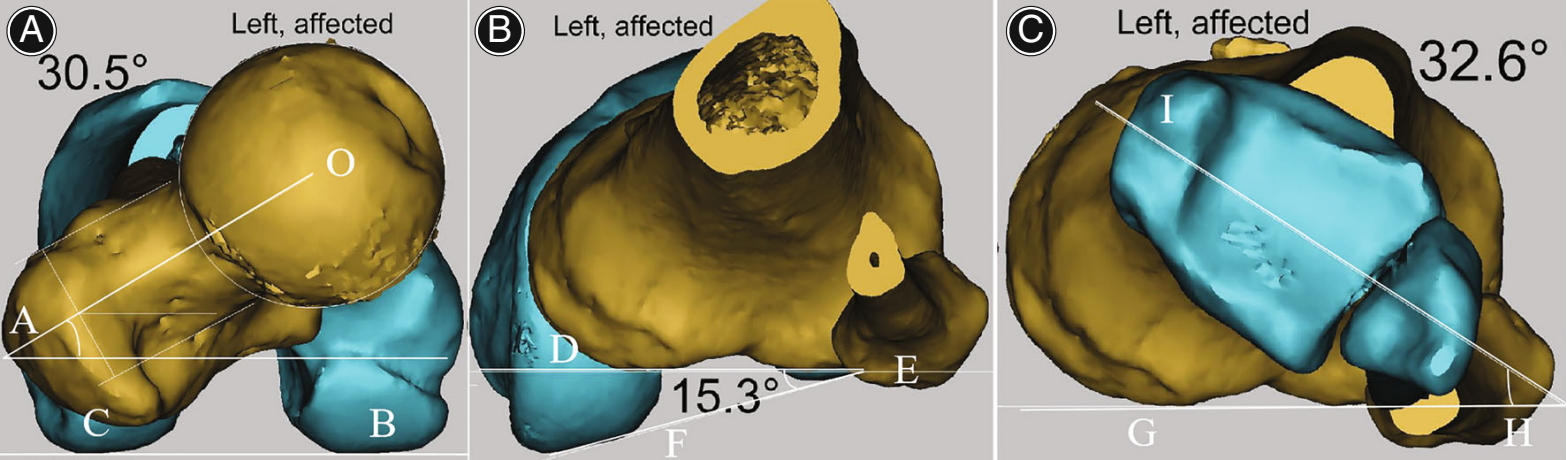
Measurement of rotational parameters of the lower extremity. (A), femoral anteversion angle is defined as the angle formed between the axis of the femoral neck and distal femur. (B), tibiofemoral rotation angle is measured by comparing the angle between the posterior femoral and tibial condylar lines. (C), tibial torsion angle is assessed by measuring the rotational angle of the proximal tibia relative to the distal tibia.

CT scans were performed on an Aquilion One scanner (Toshiba Medical Systems, America) for all patients with RPD during a maximum quadriceps contraction preoperatively. The Digital Imaging and Communications in Medicine data from the hip‐knee‐ankle CT scan were reconstructed into three‐dimensional models with Mimics Research 20.0 (Materialize, Belgium) to measure the rotational parameters of the lower extremity according to the method described previously^19^. The femoral anteversion angle is defined as the angle formed between the axis of the femoral head–neck and distal femur. Tibiofemoral rotation angle is measured by comparing the angle between the posterior femoral and tibial condylar lines. A positive angle indicates internal rotation of the distal femur relative to the proximal tibia. The tibial torsion angle is assessed by measuring the rotational angle of the proximal tibia relative to the distal tibia (Figure 2). The inter‐ and intro‐observer interclass correlation coefficient (ICC) were 0.92 and 0.91 for femoral anteversion angle, 0.89 and 0.88 for tibiofemoral rotation angle, 0.93 and 0.94 for tibial torsion angle.

### Radiological Measurements

The knee radiographs included the anteroposterior view and the lateral view with 30° of knee flexion. The Caton‐Deschamps Index (CDI) was used to measure patellar height, and patella alta was defined as CDI ≥1.2^21^. Trochlear dysplasia was detected on the true lateral view of the knee and classified according to the Dejour classification system^22^.

### Tibial Tubercle‐Trochlear Groove (TT‐TG) and Tibial Tubercle‐Posterior Cruciate Ligament (TT‐PCL) Measurement

CT scans were performed for all patients with RPD during a maximum quadriceps contraction preoperatively. All patients were examined with computerized axial tomography for the measurement of the TT‐TG distance according to the method described by Camp *et al*.^23^. The TT‐TG is evaluated by measuring the distance between the most anterior point of the tibial tuberosity and the deepest point of the trochlear groove using two lines drawn perpendicular to the tangent to the posterior borders of the femoral condyles. TT‐TG distance of more than 20 mm is believed to be nearly always associated with patellar instability. An alternative method for assessing tibial tubercle position was proposed measuring the distance in reference to the posterior cruciate ligament and not to the trochlea (TT‐PCL)^24^, which reflected the true lateralization of the tibial tubercle relative to the proximal tibia, with proposed pathologic threshold of 24 mm.

To further clarify the complicated relationship among TT‐TG, TT‐PCL, tibiofemoral rotation, and patellar tracking, patients were divided into four subgroups according to TT‐TG and TT‐PCL (Group A: TT‐TG < 20 mm, TT‐PCL < 24 mm; Group B: TT‐TG < 20 mm, TT‐PCL > 24 mm; Group C: TT‐TG > 20 mm, TT‐PCL < 24 mm; Group D: TT‐TG > 20 mm, TT‐PCL >24 mm).

### Statistical Analyses

Statistical analyses were performed with the SPSS 20.0 software package (IBM, Chicago, US). Pearson's chi‐square test or Fisher's exact test was used to compare categorical variables. For comparisons of continuous variables, Student's *t*‐test or Mann–Whitney U‐test was used. The Pearson correlation (normal data) or Spearman's rank correlation coefficient (non‐normal data) was calculated. A comparison of patellar maltracking was performed between the rotation group and control group. *p* values <0.05 were considered significant and all *p* values were two‐tailed.

## Results

### General Results

A total of 143 knees in 118 patients with RPD were included in the present study. One hundred and four patients experienced patellar dislocations during activities of daily living and 14 patients with competitive sports. No patients showed limited knee ROM preoperatively.

### Relationship between Patellar Maltracking and Bony Deformities

The mean tibiofemoral rotation angle was 12° ± 6° (range, 0°–31°) (Table 1). Pearson correlation between patellar maltracking parameters (BOI, lateral tilt) and various bony deformities found that the tibiofemoral rotation was moderately correlated with patellar tilt angle (*r* = 0.462, *p* < 0.001) and BOI (*r* = 0.451, *p* < 0.001) (Table 2).

**TABLE 1:** Demographic information of the included patients^a^

Variate	Mean ± SD (range)
Age	20.8 ± 5.8 (16–38)
Femoral anteversion	28 ± 11 (4–70)
Tibiofemoral rotation	12 ± 6 (0–31)
Tibial torsion	30 ± 8 (8–55)
TT‐TG	20.1 ± 3.9 (10.3–32.6)
TT‐PCL	23.0 ± 2.4 (12.1–31.2)
Patella height	1.19 ± 0.21 (0.73–1.78)
Trochlear dysplasia (normal/type A/ B/ C/ D)	9/64/53/3/14
J‐sign (positive/negative)	103/40

^a^ Abbreviations: SD, standard deviation; TT‐TG, tibial tuberosity‐trochlear groove; TT‐PCL, tibial tubercle‐posterior cruciate ligament.

**TABLE 2:** Pearson correlation between patellar maltracking and bony deformities^a^

Variate	BOI		Patellar tilt angle	
	r	*p* value	r	*p* value
Femoral anteversion	0.289	**0.002**	0.264	**0.004**
Tibial torsion	0.063	0.499	0.129	0.166
Tibiofemoral torsion	0.451	**<0.001**	0.462	**<0.001**
Patellar height	0.306	**0.001**	0.192	**0.044**
TT‐TG	0.548	**<0.001**	**0.559**	**<0.001**
TT‐PCL	0.143	0.235	0.097	0.321

^a^ Abbreviations: BOI, bisect offset index. bolded values indicate statistical significance (*p* < 0.05); TT‐TG, tibial tuberosity‐trochlear groove; TT‐PCL, tibial tubercle‐posterior cruciate ligament.

### Association between Tibiofemoral Rotation and Patellar Maltracking

The association between tibiofemoral rotation and patellar maltracking was further analyzed. Results demonstrated that the BOI (152.1 *vs* 121.2, *p* < 0.001) and patellar tilt angle (41.2° *vs* 33.5°, *p* < 0.001) were significantly higher in the rotation group (tibiofemoral rotation≥15°) than that in control group (tibiofemoral rotation<15°) (Table 3).

**TABLE 3:** Comparison results between rotation group and control group

Variables	Rotation group	Control group	Statistic value
Age	21.2 ± 6.7	20.7 ± 5.4	*t* = 0.497, *p* = 0.620
Gender (F/M)	31/6	90/16	*χ* ^*2*^ = 0.027, *p* = 0.871
Femoral anteversion	29 ± 13	27 ± 10	*t* = 1.033, *p* = 0.303
Tibial torsion	29 ± 7	30 ± 9	*t* = −1.552, *p* = 0.123
Tibiofemoral Rotation	19 ± 3	9 ± 4	***t* = 13.970, *p* < 0.001**
Patella height	1.27 ± 0.23	1.16 ± 0.20	***t* = 2.664, *p* = 0.009**
Severe trochlear dysplasia	22	44	*χ* ^*2*^ = 3.556, *p* = 0.059
TT‐TG	21.9 ± 4.2	19.4 ± 3.6	***t* = 3.529, *p* = 0.001**
TT‐PCL	21.2 ± 3.2	23.6 ± 2.2	***t* = 2.354, *p* = 0.021**
BOI	152.1 ± 46.8	121.2 ± 35.7	***t* = 3.851, *p* = 0.001**
Patellar tilt angle	41.2 ± 12.2	33.5 ± 10.4	***t* = 3.439, *p* = 0.001**

Abbreviations: BOI, bisect offset index; bolded values indicate statistical significance (*p* < 0.05); F/M, female/male; TT‐TG, tibial tuberosity‐trochlear groove; TT‐PCL, tibial tubercle‐posterior cruciate ligament.

### Comparison among the Four Subgroups

The results of comparison among the four subgroups were showed in Table 4. For patients with a TT‐TG distance of < 20 mm, no significant difference was found in BOI (117 *vs*109, *t = 1.012, p* = 0.315) and patellar tilt angle (31.7 *vs* 29.1, *t = 0.399, p* = 0.691) between these two subgroups. For patients with a TT‐TG distance of >20 mm, Group C had normal TT‐PCL and increased tibiofemoral rotation, in contrast, Group D had increased TT‐PCL and relatively normal tibiofemoral rotation. In other words, the increased TT‐TG was mainly caused by tibiofemoral rotation angle in Group C and was predominantly induced by tibial tubercle lateralization in Group D. Further comparison showed that the BOI (159 *vs* 127, *t = 13.764, p* < 0.001) and patellar tilt angle (43.0 *vs* 36.6, *t = 5.643, p* < 0.001) were significantly higher in the Group C than Group D.

**TABLE 4:** Subgroup analysis results based on TT‐TG and TT‐PCL^a^

	TT‐TG < 20 mm		Statistic value	TT‐TG > 20 mm		Statistic value
	TT‐PCL < 24	TT‐PCL > 24		TT‐PCL < 24	TT‐PCL > 24	
Number	41	27		43	32	
Age	20.9	20.1	*t = 0.648, p* = 0.519	21.2	20.7	*t = 0.485, p* = 0.629
Gender (F/M)	35/6	22/5	*χ* ^*2*^ *= 0.181, p* = 0.743	38/5	26/6	*χ* ^*2*^ *= 0.743, p* = 0.513
Affected sige (L/R)	24/17	15/12	*χ* ^*2*^ *= 0.059, p* = 0.808	21/22	15/17	*χ* ^*2*^ *= 0.028, p* = 0.866
TT‐TG	16.9 ± 2.6	17.3 ± 1.7	*t = −0.370, p* = 0.712	22.8 ± 3.2	22.9 ± 2.5	*t = −0.137, p* = 0.891
TT‐PCL	20.3 ± 2.9	26.4 ± 2.3	***t = −4.137, p* < 0.001**	21.0 ± 3.6	26.4 ± 2.0	***t = 5.783, p* < 0.001**
Tibiofemoral Rotation	12 ± 6	6 ± 4	***t = 12,456, p* < 0.001**	15 ± 5	11 ± 5	***t = 7451, p* < 0.001**
Femoral Anteversion	28 ± 9	27 ± 10	*t = 0.993, p* = 0.324	29 ± 15	25 ± 9	*t = 1.473, p* = 0.145
Tibial Torsion	30 ± 8	29 ± 7	*t = 0.538, p* = 0.592	29 ± 7	31 ± 9	*t = −1.247, p* = 0.216
CDI	1.13 ± 0.19	1.16 ± 0.17	*t = −1.241, p* = 0.219	1.20 ± 0.22	1.22 ± 0.20	*t = −1.077, p* = 0.285
Severe TD, n (%)	17 (41%)	8 (30%)	*χ* ^*2*^ *= 0.980, p* = 0.322	24 (56%)	16 (50%)	*χ* ^*2*^ *= 0.249, p* = 0.618
BOI	117 ± 38	109 ± 16	*t = 1.012, p* = 0.315	159 ± 51	127 ± 24	***t = 13.764, p* < 0.001**
Patellar Tilt angle	31.7 ± 11.7	29.1 ± 7.4	*t = 0.399, p* = 0.691	43.0 ± 11.3	36.6 ± 7.9	*t = 5.643, p* **< 0.001**

^a^ Abbreviations: BOI, bisect offset index; bolded values indicate statistical significance (*p* < 0.05); TT‐TG, tibial tuberosity‐trochlear groove; TT‐PCL, tibial tubercle‐posterior cruciate ligament; CDI, Caton–Deschamps index; TD, trochlear dysplasia.

## Discussion

### Main Findings of this Study

The most important finding of this study is that the increased tibiofemoral rotation angle is associated with patellar maltracking in patients with RPD. Tibiofemoral rotation‐induced TT‐TG abnormality deserves more attentions in clinical practice, because these patients usually have more serious patellar maltracking compared with tibial tubercle lateralization‐caused TT‐TG abnormality.

### Influence of Tibiofemoral Rotation on Patellar Tracking

Tibiofemoral rotation angle, as an important component of rotational parameters of the lower extremity, has been found to be higher in patients with RPD than that in healthy population^16^, ^18^, ^19^, ^25^, which indicates that an increased tibiofemoral rotation angle may be a potential risk factor for RPD. However, few studies have investigated the association between the increased tibiofemoral rotation and patellar maltracking in the literature. Lin *et al*. compared the degree of tibiofemoral rotation in different populations and found that tibiofemoral rotation was correlated with the severity of patellar dislocation, with the greatest value in fixed or obligatory dislocation patients^13^. Keshmiri *et al*. analyzed the influence of rotational limb alignment parameters on patellar kinematics, which demonstrated that tibiofemoral rotation produced a significant influence on patellar medial‐lateral shift in cadaveric knees^20^. Consistent with the study of Keshmiri, the present study found that the tibiofemoral rotation was moderately correlated with the patellar tilt angle and BOI, but with one main difference: this present research was conducted in patients with RPD but not in normal cadaveric knees. Based on the above results, one possibility, far from proved, is that the increased tibiofemoral rotation angle is a contributing factor for patellar maltracking in patients with RPD. However, it must be noted that patella height was significantly higher in the rotation group than the control group as described in Table 3, the patella alta might be as much the origin for patellar maltracking as the tibiofemoral rotation, and further studies are needed to demonstrated the complex relationship between tibiofemoral rotation and patellar maltracking.

### Relationship between TT‐TG and Tibiofemoral Rotation

TT‐TG distance has long been thought to reflect the lateralization of the tibial tubercle and is commonly used as an indicator for tibial tubercle medicalization. However, recent studies found that the TT‐TG was in fact an amalgamated measure of true lateralization of the tibial tubercle and the tibiofemoral rotation^18^, ^24^, ^26^. Tensho *et al*. found that TT‐TG distance had no linear correlation with tibial tubercle lateralization^18^, ^27^, however, tibiofemoral rotation strongly correlated with the TT‐TG distance (r = 0.69) in patients with RPD, therefore, they concluded that TT‐TG distance was affected more by tibiofemoral rotation than by tibial tubercle lateralization; therefore, its use as an indicator for tibial tubercle transfer might be inappropriate. Similarly, Anley *et al*. also declared that an increased TT‐TG may result from the true lateralization of the tibial tubercle, an increased tibiofemoral rotation, or both^24^. However, if a tibial tubercle medialization is performed in a patient without major tubercle lateralization (such as patients with a TT‐TG > 20 mm, but TT‐PCL < 24 mm), the patellar tracking may not normalize, and abnormal stress on the medial patellofemoral joint may persist because the underlying cause of increased TT‐TG distance in these patients has not been settled precisely. Parikh *et al*. stated that tibial tubercle osteotomy could increase external torsion of the tibia and exacerbate symptoms in the presence of underlying rotational malalignment^28^. Nakagawa *et al*. reported the long‐term results of tibial tubercle osteotomy in 39 patients and found that the osteoarthritic changes of the patellofemoral joint had advanced in up to 42% of patients^29^. Therefore, surgeons should recognize that this procedure may not always represent a patient‐centered treatment in some patients who have a TT‐TG elevated by knee rotation and not by lateralization of the tibial tubercle. In these patients, it may be appropriate to use another technique (e.g. derotational osteotomy, trochleoplasty) that is based on individual pathological conditions. Thus, indications for tibial tubercle medialization for the treatment of RPD should not merely be determined by the TT‐TG distance, but also another value that directly reflects tubercle lateralization at the proximal part of the tibia, such as the TT‐PCL distance.

### Correlation between TT‐TG and Patellar Maltracking

The correlation between TT‐TG and patellar maltracking has been reported. Williams *et al*. evaluated the role of TT‐TG distance in patellofemoral kinematics using dynamic CT and found that an association existed between TT‐TG distance and the patellar tracking parameters^30^, which was in line with the present study. To further clarify the complicated relationship among TT‐TG, TT‐PCL, tibiofemoral rotation, and patellar maltracking, patients were divided into four subgroups. The most interesting finding is that if the increased TT‐TG distance is mainly caused by abnormal tibiofemoral rotation, which accounting for 57% of all cases with increased TT‐TG in the present study, patients usually have severe patellar maltracking, which further suggested the obvious correlation between tibiofemoral rotation and patellar maltracking in RPD.

### The Limitations of the Study

There were several limitations of this study. First, patellar maltracking was assessed using static CT measurements, which may underestimate or overestimate the severity of the true patellar maltracking. Second, in the present study, patients are divided into rotation group (tibiofemoral rotation≥15) and control group (tibiofemoral rotation<15), which is without theoretical justification because little research on this has yet been done and further studies are needed to confirm what is the threshold of pathological tibiofemoral rotation angle. Third, although the present study found a potential correlation between tibiofemoral rotation and patellar maltracking, we did not further investigate the potential causes of increased tibiofemoral rotation and how to correct this rotational deformity.

### Conclusion

The increased tibiofemoral rotation angle is associated with patellar maltracking in patients with recurrent patellar dislocation. Patients with increased tibiofemoral rotation angle usually have more severe patellar maltracking. Therefore, tibiofemoral rotation angle should be measured routinely preoperatively to assess the patellar tracking more accurately.

## Funding

This study was funded by Capital's Funds for Health Improvement and Research (2020–1‐2075) and Beijing JST Research Funding (QN202202).

## Conflicts of Interest

The authors declare that they have no conflict of interest.

## Authors Contributions

Guan Wu participated in study design, data collection, and drafted the manuscript. Hui Zhang and GuanYang Song carried out the radiological measurements. Tong Zheng participated in the data collection and statistical analysis. YanWei Cao carried out the radiological measurements. Yue Li participated in the study design and data collection. Zhi Jun Zhang conceived of the study, and participated in its design and helped to draft the manuscript. All authors read and approved the final manuscript.

## Ethics Approval

All procedures performed in this retrospective study were in accordance with the ethical standards of the Beijing Jishuitan hospital, and this study was performed after obtaining approval from our institutional review board (IRB, No. 20200701).

## Consent to Participate

All patients provided informed consent before participating in this study.

## Consent for Publication

All patients provided informed consent for publication.

## Availability of Data and MateriaL

The datasets used or analyzed during the current study are available from the corresponding author on reasonable request

## Code Availability

Yes
